# Kwaliteitsverbetering als een gezamenlijke opgave: Evaluatie van een kwaliteitsinstrument in de verpleeghuiszorg

**DOI:** 10.1007/s12508-023-00390-1

**Published:** 2023-05-04

**Authors:** Juultje Naber, Yael Reijmer, Bellis van den Berg

**Affiliations:** Vilans, landelijke kennisorganisatie voor zorg en ondersteuning, Utrecht, Nederland

**Keywords:** kwaliteitskader verpleeghuiszorg, kwaliteitsverbetering, procesevaluatie, scan, Nursing home care quality framework, Quality improvement, Process evaluation, Scan

## Abstract

Om de implementatie van het Kwaliteitskader Verpleeghuiszorg in de praktijk te bevorderen startte het ministerie van Volksgezondheid, Welzijn en Sport (VWS) in 2019 in samenwerking met kennisorganisatie Vilans het ondersteuningsprogramma Waardigheid en trots op locatie. Verpleeghuislocaties die deelnemen aan dit programma starten met een Scan Kwaliteitskader Verpleeghuiszorg (scan KKV), die in kaart brengt wat de kwaliteit van zorg is ten opzichte van het kwaliteitskader en welke verbeterpunten aandacht behoeven. Dit artikel beschrijft de werkzame elementen van deze scan en de geleerde lessen.

**Digitaal aanvullende content:**

De online versie van dit artikel (10.1007/s12508-023-00390-1) bevat aanvullend materiaal, toegankelijk voor daartoe geautoriseerde gebruikers.

## Abstract (en)

This article presents the key elements of the quality measure included in the Dignity and Pride program of the Dutch Ministry of Health, Welfare and Sport in cooperation with Vilans, the national centre of expertise for long-term care in the Netherlands. Nursing homes take part in the quality measure at the start of the program to investigate where they stand with respect to the nursing home quality framework.

## Inleiding

Sinds de invoering van de Zorgverzekeringswet in 2006 is er ingezet op transparantie ten aanzien van kwaliteit van zorg. Verschillende indicatoren en metingen zijn ontwikkeld om de kwaliteit inzichtelijk te maken voor zorgverzekeraars (inkoopinformatie), patiënten en cliënten (keuze-informatie), de Inspectie Gezondheidszorg en Jeugd (IGJ; toezicht) en zorgorganisaties (verbeterinformatie). Voor verpleeghuizen betekende dit dat zij jaarlijks een set aan zorginhoudelijke indicatoren aanleverden en tweejaarlijks cliëntervaringen ophaalden met behulp van de Consumer Quality index (CQi) [1]. De informatie die dit opleverde bleek niet voor alle groepen even bruikbaar. Cliëntenervaringen werden teruggebracht naar een gemiddelde en verpleeghuisorganisaties en medewerkers gaven aan dat de indicatoren niet altijd de werkelijke kwaliteit van zorg representeerden.

De nadruk op verantwoording en metingen gebaseerd op indicatoren en de daarmee gepaard gaande toename aan registraties en regels werden gezien als verantwoording afleggen aan externe partijen, waarbij betekenisvolle informatie voor verbeteren van de zorg werd gemist [2]. De ervaringen uit deze periode hebben ons geleerd dat kwaliteit niet statisch en objectief meetbaar is, maar (inter)subjectief en afhankelijk van tijd en plaats is [3]. Ook is duidelijk dat er voor leren en verbeteren méér nodig is dan een jaarlijkse of tweejaarlijkse meting op organisatieniveau. Om daadwerkelijk aan de slag te kunnen gaan met kwaliteitsverbetering is het wenselijk om verschillende perspectieven mee te nemen en gebruik te maken van actuele inzichten, is er ruimte nodig voor een open dialoog en moeten medewerkers worden begeleid en eigenaarschap ervaren wat betreft het verbeterproces [3, 4].

Met de komst van het nieuwe Kwaliteitskader Verpleeghuiszorg in 2017, waarin staat beschreven wat de cliënt van de zorg mag verwachten en wat daarvoor nodig is [5], is de focus verlegd naar gezamenlijke kwaliteitsverbetering met alle betrokken partijen. Sindsdien is een groot aantal instrumenten ontwikkeld en in gebruik genomen om (ervaren) kwaliteit inzichtelijk te maken en te verbeteren. Deze instrumenten variëren van vragenlijsten tot narratief onderzoek en observaties [4, 6, 7], waarbij is geconcludeerd dat enkel rapporteren op norm en getal niet voldoende is om de complexiteit van de langdurige zorg in beeld te brengen [2, 3]. Inmiddels worden kwantitatieve gegevens steeds vaker gecombineerd met kwalitatieve informatie en dialoogvormen waarin ervaringen rond de kwaliteit van zorg worden gedeeld [4, 7].

## Het Kwaliteitskader Verpleeghuiszorg: samen leren en ontwikkelen

Vanuit het ministerie van Volksgezondheid Welzijn en Sport (VWS) wordt al sinds geruime tijd via verschillende programma’s ingezet op de verbetering van de kwaliteit in de langdurige zorg. In de periode 2005–2019 heeft kennisorganisatie Vilans in dit kader de programma’s Zorg voor beter, In voor zorg! en Waardigheid en trots uitgevoerd [8].

Om de implementatie van het Kwaliteitskader Verpleeghuiszorg in de praktijk te bevorderen startte het ministerie van VWS, in 2019 in samenwerking met kennisorganisatie Vilans, het ondersteuningsprogramma Waardigheid en trots op locatie. In dit programma werd voortgebouwd op de ervaringen en geleerde lessen uit de voorgaande programma’s. Nieuw is dat verpleeghuislocaties die aan dit programma deelnemen met een Scan Kwaliteitskader Verpleeghuiszorg (scan KKV) starten, waarmee in kaart wordt gebracht en het gesprek wordt gestart over wat de kwaliteit van zorg is ten opzichte van het kwaliteitskader. Hierbij worden vanuit verschillende perspectieven ervaringen opgehaald en wordt besproken welke punten voor verbetering vatbaar zijn. De scan KKV wordt tussentijds en aan het einde van de deelname aan het programma herhaald om inzicht te krijgen in de ontwikkeling van de kwaliteit op een locatie.

### De Scan Kwaliteitskader Verpleeghuiszorg (scan KKV): werkwijze

De scan KKV combineert een aantal aspecten die van belang zijn bij het inzichtelijk maken van de (ervaren) kwaliteit, het leren hiervan en het verbeteren van de kwaliteit. Het proces betreft een aantal stappen waarmee wordt toegewerkt naar een gezamenlijk beeld van de locatie, op basis van de acht thema’s van het Kwaliteitskader Verpleeghuiszorg. De acht thema’s zijn: 1) persoonsgerichte zorg en ondersteuning, 2) wonen en welzijn, 3) veiligheid, 4) leren en verbeteren van kwaliteit, 5) leiderschap, governance en management, 6) personeelssamenstelling, 7) gebruik van hulpbronnen, en 8) gebruik van informatie. Het proces bestaat uit de volgende drie stappen:

1. De vragenlijst (zie de bijlage) wordt afgenomen bij cliënten, naasten, (zorg)medewerkers, het management en het bestuur. De respondenten kunnen op een schaal van 1 (helemaal niet mee eens) tot 5 (helemaal mee eens) aangeven in hoeverre ze het eens zijn met verschillende stellingen over de acht thema’s van het Kwaliteitskader. Ook is er ruimte voor een toelichting. De formulering van de stellingen is aangepast en afgestemd op de (functie)groep [9].
2. De uitkomsten van de vragenlijst worden geanalyseerd door twee externe, onafhankelijke experts (de zogenaamde ‘scanners’). Deze kennen een score toe aan elk van de onderwerpen die bij de acht kwaliteitsthema’s behoren. De scoring volgt het stoplichtmodel, variërend van rood (ernstige problemen) tot donkergroen (uitblinker). Bij het toekennen van de scores wordt gelet op eventuele discrepanties tussen de ervaringen van (verschillende groepen) respondenten, toelichtingen bij de vragen en informatie uit andere beschikbare documenten, zoals audits en IGJ-rapporten. Ten slotte wordt gekeken in hoeverre het beeld dat hieruit ontstaat overeenkomt met de landelijke kwaliteitsnormen (het kwaliteitskader). Onderwerpen waar geen of weinig consensus over bestaat óf die overwegend negatief dan wel positief worden beoordeeld komen in aanmerking voor het kwaliteitsgesprek.
3. Een kwaliteitsgesprek waarin de voorlopige interpretatie van de uitkomsten van de vragenlijst wordt getoetst bij vertegenwoordigers van verschillende functiegroepen en cliëntenvertegenwoordigers. De deelnemers reflecteren op de uitkomsten, duiden deze en trekken gezamenlijk conclusies over de definitieve themascores. Het kwaliteitsgesprek wordt begeleid door de twee scanners.

De gezamenlijk vastgestelde scores worden verwerkt in een rapport en gevisualiseerd. De uitslag wordt vervolgens teruggekoppeld aan het bestuur en de overige functiegroepen. Als het bestuur zich aan de uitslag committeert wordt er, indien nodig en gewenst, een ondersteuningstraject gestart. Dat duurt een tot twee jaar en wordt begeleid door een coach. In de praktijk is er bij 87 % van de locaties gekozen voor het starten van een leer- en verbetertraject met ondersteuning vanuit het programma Waardigheid en trots op locatie.

De scan is inmiddels bij ruim 500 verpleeghuislocaties (circa 25 % van het totale aantal verpleeghuislocaties in Nederland) een of meer keren uitgevoerd. De scan kan periodiek worden herhaald om de kwaliteit te monitoren, opnieuw de dialoog te voeren en nieuwe prioriteiten te stellen voor kwaliteitsverbetering.

## Evaluatie van het scanproces

Vilans deed onderzoek naar de ervaringen met de scan KKV op locaties, met als doel inzicht te krijgen in de werkzame elementen en geleerde lessen. Voor het onderzoek zijn zestien interviews afgenomen aan de hand van een topiclijst bij locatiemedewerkers (N = 10) en bij scanners die de scan KKV hebben uitgevoerd (N = 6). Sommige locatiemedewerkers werkten op meerdere locaties, waardoor er informatie is opgehaald over in totaal 26 locaties van verschillende grootte, verspreid over het land. De locatiemedewerkers hadden verschillende functies, zoals teamleider, bestuurder en kwaliteitsverpleegkundige. De semigestructureerde interviews zijn afgenomen door twee onderzoekers in wisselende samenstellingen, waarbij één onderzoeker notuleerde en de ander het gesprek voerde. De gespreksverslagen zijn door één onderzoeker op onderwerp gecodeerd, wat is geverifieerd door een tweede onderzoeker.

## Resultaten en beschouwing

De betrokken locatiemedewerkers en scanners waren over het algemeen positief over de opbrengst van de scan. Zij waardeerden met name het inzicht dat de scan geeft in waar de locatie staat ten opzichte van de normen in het kwaliteitskader, dat zorgprofessionals zich na afloop meer bewust zijn van de kwaliteitsnormen, de vergroting van het urgentiebesef om bepaalde zaken te verbeteren en het voeren van het gesprek over kwaliteit met cliënten en tussen functiegroepen. Alle drie de onderdelen van de scan droegen hier volgens hen aan bij en kunnen dan ook niet los van elkaar worden gezien. Hieronder bespreken we de belangrijkste werkzame elementen en de beperkingen van de scan.

### De werkzame elementen en geleerde lessen

#### Het uitvragen van verschillende perspectieven geeft een breed beeld van de ervaren kwaliteit van de zorg en creëert draagvlak voor een vervolg

De geïnterviewden benadrukken dat het belangrijk is om alle (functie)groepen erbij te betrekken en voldoende respons op te halen. Ook vinden ze van belang dat de vragenlijst ingaat op alle onderdelen van het kwaliteitskader. Hiermee worden de verschillende onderdelen vanuit de verschillende perspectieven geëvalueerd en ontstaat er een compleet beeld van de ervaringen met betrekking tot de onderwerpen.

Een bijkomstig werkzaam element is dat het invullen van de vragenlijst het startpunt vormt van een bewustwordingsproces over de kwaliteit van de zorg. Door de vragenlijst breed uit te zetten wordt er al vanaf het begin een grote groep medewerkers bij betrokken, die hun stem kunnen laten horen door aan te geven hoe zij de verschillende onderdelen van het kwaliteitskader op de locatie ervaren. Volgens de geïnterviewden was goede communicatie over het doel van de scan en het mogelijke verbetertraject hiervoor een belangrijke voorwaarde, net zoals het informeren van medewerkers over de uitkomsten van de scan en het betrekken van medewerkers bij het mogelijke vervolgtraject. Een locatiemanager vertelde: ‘Wat hielp: veel informatie verstrekken en veel uitleggen. Die inspanningen hebben zeker wat opgeleverd.’ Ook werd het belang van het gebruik van simpele taal onderstreept. In sommige gevallen heeft de locatie vrijwilligers ingeschakeld om cliënten te helpen bij het lezen en invullen van de vragenlijsten.

Bij het invullen van de vragenlijst werd er volop gebruikgemaakt van de ruimte om toelichting te geven bij de reactie op de stellingen. Deze toelichting geeft kleur aan de scores van de respondent en wordt gebruikt door de scanners als input voor het kwaliteitsgesprek. Het gebruik van een vragenlijst heeft als beperking dat niet kan worden doorgevraagd en dat de vraag niet zoals bij een interview kan worden verduidelijkt. Zeker bij de doelgroep van cliënten en naasten kan dit relevant zijn. Tijdens het kwaliteitsgesprek was er wel ruimte om te verduidelijken en door te vragen.

#### Het analyseren van de uitkomsten van de vragenlijst en overige bronnen geeft inzicht in belangrijke overeenkomsten en verschillen

Voor de analyse beschikken de scanners over de uitkomsten van de vragenlijsten, de toelichting die respondenten bij hun scores gaven en een aantal belangrijke documenten (zoals IGJ-rapporten, cliënttevredenheidsonderzoek en jaarverslagen). Door informatie uit deze verschillende bronnen en van verschillende perspectieven samen te nemen wordt gepoogd een zo volledig mogelijk beeld te vormen van de kwaliteit van de zorg.

**Figure 1:**
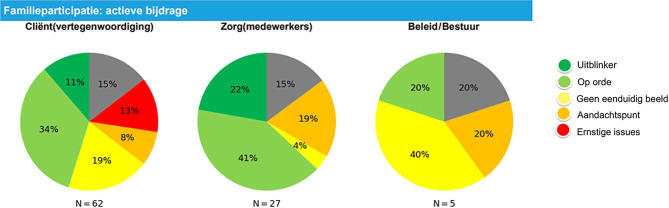

De scanners gaven aan dat de analyse van deze bronnen veel tegenstellingen aan het licht brengt. Zo kwam het geregeld voor dat medewerkers veel kritischer waren dan het management of dat cliënten opvallend lager scoorden dan medewerkers (zie fig. 1). In deze gevallen prioriteerden de scanners het onderwerp voor het kwaliteitsgesprek om de reden van het verschil boven tafel te krijgen.

Andere redenen om een onderwerp voor het kwaliteitsgesprek te prioriteren waren opvallend positieve of negatieve beoordelingen, uitingen van onvrede in de toelichting bij de stellingen (die soms niet overeenkwamen met de gegeven score) of een plotselinge toename van het aantal keren dat het antwoord ‘onvoldoende zicht op’ werd gegeven (bijvoorbeeld een stelling over leiderschap die door een deel van de medewerkers werd overgeslagen). De scanners vonden de visualisatie van de scores prettig omdat deze hen hielp om opvallende patronen eenvoudig uit de grote hoeveelheid informatie te halen en zo goed toegerust het gesprek aan te gaan.

#### Het kwaliteitsgesprek vergroot de bewustwording en leidt tot een gezamenlijk perspectief

Het reflecteren op kwaliteit en het uitwisselen van ervaringen tijdens het kwaliteitsgesprek (stap 3) werden door de geïnterviewden als erg waardevol ervaren en leidden tot nieuwe inzichten bij de aanwezigen. Er ontstond in de eerste plaats een completer beeld van de situatie op de locatie. In de meeste gevallen werd men tijdens het gesprek ook kritischer over het eigen handelen: medewerkers werden meer ‘bewust onbekwaam’.

Niet alleen de verschillende ervaringen, maar ook de inbreng van kennis over de landelijke normen door de scanners droeg bij aan het vergroten van het kwaliteitsbewustzijn. Meerdere deelnemers gaven aan dat het spiegelen aan het kwaliteitskader ervoor zorgde dat ze op een andere manier naar kwaliteit gingen kijken. ‘Zoals het altijd gaat op de locatie’ is dan niet meer de norm.

Maar ook als ze zich wél bewust waren van de eigen tekorten, of als medewerkers zelf de lat erg hoog legden, ervaarden ze het als prettig om dat bevestigd te zien in de uitkomst van de scan. Het gezamenlijke perspectief op kwaliteit vormde een breed gedragen uitgangspunt voor het leer- en verbeterproces.

Een enkele keer werd er tijdens het drie uur durende gesprek geen consensus bereikt over de score van een onderwerp. Dit werd dan aangegeven in het verslag en verder opgepakt tijdens de uitvoering van het verbetertraject.

#### Externe procesbegeleiders zorgen voor sturing en een kritische blik

Het mag niet onbenoemd blijven dat de scanners een essentiële rol spelen bij het verloop van het scanproces. Volgens de geïnterviewden was het van grote meerwaarde dat de scanners het proces begeleidden, het tijdspad bewaakten en medewerkers betrokken hielden bij het proces. Anders was de kans groot geweest dat ze werden opgeslokt door de waan van de dag en dat het scanproces niet volledig werd doorlopen of zou vertragen. Ook zorgden de scanners tijdens het kwaliteitsgesprek voor een veilige en open sfeer waarin iedereen zich kan uitspreken en wordt gehoord. Ze droegen bij aan de bewustwording door kritische vragen te stellen, een spiegel voor te houden en kennis in te brengen over kwaliteitsnormen.

Het bleek hierbij een voordeel dat de scanner geen onderdeel uitmaakt van de organisatie. Meerdere geïnterviewden gaven aan dat een ‘blik van buiten’ helpt bij het ontdekken van blinde vlekken en het overstijgend analyseren van de situatie om zo sneller tot de kern van het probleem te komen. Ook konden de scanners gebruikmaken van hun ervaringen en geleerde lessen op andere locaties.

Een belangrijk aandachtspunt hierbij was dat de scanner niet de rol innam van adviseur, maar dat de locatie zelf tot een definitief eindoordeel moest komen. Een scanner zei: ‘Je moet gewoon nieuwsgierig zijn naar de [ervaringen van] deelnemers, naar het verhaal. Geen oordeel hebben. Niet spreken vanuit een coachrol of adviesrol.’

### Beperkingen

De scan KKV kent ook een aantal beperkingen. Zoals eerder gezegd is kwaliteit een dynamisch concept, afhankelijk van persoon, tijd en plaats [3]. De uitkomst van de scan is daarmee afhankelijk van wie er aan tafel zit en wat er op dat moment op de locatie speelt. Dit onderstreept dat het belangrijk is dat de vragenlijst wordt ingevuld door een groot aantal respondenten en verschillende stakeholders aan het kwaliteitsgesprek deelnemen. Hiermee kan een te eenzijdig of gekleurd beeld worden voorkomen. Om die reden wordt bij het kwaliteitsgesprek gestreefd naar een divers gezelschap van circa tien personen, waarbij de cliënt, de (zorg)medewerker en het management vertegenwoordigd zijn. De ervaring leert inmiddels dat dit kwalitatief de beste gesprekken oplevert.

Het tijdsbestek (3 uur) beperkt het aantal onderwerpen dat tijdens het kwaliteitsgesprek kan worden besproken en de mate waarin dieper op elk onderwerp kan worden ingegaan. De geïnterviewden vonden dat het kwaliteitsgesprek een flinke tijdinvestering vergde, maar achtten het ook zeker de moeite waard. De analyse van de vragenlijsten en beschikbare bronnen vormt een belangrijke stap om tot een effectieve en onderbouwde selectie van de gespreksonderwerpen te komen.

Ten slotte is het onduidelijk in hoeverre het reflecteren op kwaliteit na afloop van de scan wordt geborgd. Een aantal locaties gaf aan dat (door de COVID-19-crisis) de aandacht was verslapt. Andere locaties kiezen ervoor om de scan periodiek (bijvoorbeeld ieder jaar) uit te voeren, om zo het leer- en verbeterproces onder medewerkers levend te houden en ontwikkelingen in de tijd te monitoren.

## Conclusie

De eerste stap bij het verbeteren van kwaliteit betreft bewustwording en urgentiebesef [10]. De scan KKV draagt hieraan bij doordat hij uitgaat van verschillende perspectieven en deze vervolgens spiegelt aan het kwaliteitskader en de beschikbare feitelijke (beleids)informatie van een locatie. We concluderen dat de elementen van de scan handvatten kunnen bieden voor het verbeteren van de kwaliteit in de verpleeghuissector (en mogelijk ook in andere sectoren). Het gaat hierbij om: 1) het breed ophalen van meningen, 2) het vormen van een integraal beeld van de kwaliteit (dus inhoud en randvoorwaarden), 3) het met elkaar vergelijken van perspectieven, en 4) het uitvoeren van een gedegen analyse door externen, om 5) vervolgens met (vertegenwoordigers van) de diverse betrokkenen gezamenlijk in gesprek te gaan.

Samengevat werkt de scan als een ‘vliegwiel’ dat het werken aan kwaliteit op een locatie in gang zet op basis van openheid, dialoog, bewustwording en consensus over vervolgstappen.

Het werken aan kwaliteit is echter niet afgelopen op het moment dat de scan is afgerond. Daarom is het van belang om de scan periodiek te herhalen om de focus op kwaliteit te behouden en zo nodig nieuwe prioriteiten te stellen.
