# Supplementary material for: Quality improvement as a joint task: Evaluation of a quality instrument in nursing home care
Source: TSG. 2023 May 4;101(2):58–62. [Article in Dutch] doi: 10.1007/s12508-023-00390-1 (PMC10157124; doi:10.1007/s12508-023-00390-1)
Supplement: Supplementary file 1 [file 12508_2023_390_MOESM1_ESM.docx]

Deze vragenlijst is ontwikkeld in het kader van programma Waardigheid en trots op locatie. Dit document kan worden gebruikt om inzicht te krijgen in hoe de vragenlijst is opgebouwd en ter inspiratie, maar is niet geschikt voor vrij gebruik. Mocht jouw organisatie gebruik willen maken van deze vragenlijst neem dan contact op met Vilans: [KVC@vilans.nl](mailto:KVC@vilans.nl)

| **Onderwerp** | **Subgroep** | **Definitieve Vraag** |
| --- | --- | --- |
| Thema 1: Persoonsgerichte zorg | | |
| Aandacht voor bewoners | Bestuur/(Zorg)management/Beleid en kwaliteit | Er zijn duidelijke afspraken rondom persoonsgericht werken en wij ondersteunen medewerkers om hiernaar te kunnen handelen. |
| Aandacht voor bewoners | Cliënt/Naaste | Medewerkers hebben voldoende aandacht voor mij en mijn situatie. Dat betekent dat zij persoonlijk contact maken, betrokken zijn en goed luisteren naar wat ik zeg. |
| Aandacht voor bewoners | Verzorgende/Verpleegkundige/Helpende | Medewerkers hebben voldoende aandacht voor bewoners en hun situatie. Dat betekent dat zij persoonlijk contact maken, betrokken zijn en goed luisteren naar wat bewoners zeggen. |
| Opstellen zorgleefplan | Bestuur/(Zorg)management/Beleid en kwaliteit | Er zijn duidelijke afspraken rondom het opstellen en werken met het zorgleefplan en wij ondersteunen medewerkers om hiernaar te kunnen handelen. |
| Opstellen zorgleefplan | Cliënt/naaste | Medewerkers stellen samen met mij een zorgleefplan op met doelen en afspraken. Mijn wensen, behoeften en mogelijkheden zijn daarvoor het uitgangspunt. |
| Opstellen zorgleefplan | Verzorgende/Verpleegkundige/Helpende | Medewerkers stellen samen met bewoners en/of naasten een zorgleefplan op met doelen en afspraken. De wensen, behoeften en mogelijkheden van de bewoner zijn daarvoor het uitgangspunt. |
| Zorgleefplan binnen 6 weken | Bestuur/(Zorg)management/Beleid en kwaliteit | Iedere bewoner heeft binnen 6 weken nadat hij of zij hier is komen wonen een compleet ingevuld zorgleefplan. |
| Zorgleefplan binnen 6 weken | Verzorgende/Verpleegkundige/Helpende | Iedere bewoner heeft binnen 6 weken nadat hij of zij hier is komen wonen een compleet ingevuld zorgleefplan. |
| Zorgleefplan kennen bewoner | Cliënt/Naaste | Uit mijn zorgleefplan blijkt dat medewerkers mij goed kennen en rekening houden met mijn wensen, behoeften, mogelijkheden en beperkingen. |
| Zorgleefplan kennen bewoner | Verzorgende/Verpleegkundige/Helpende | Uit de zorgleefplannen blijkt dat medewerkers de bewoners goed kennen en rekening houden met hun wensen, behoeften, mogelijkheden en beperkingen. |
| Verzorging volgens Zorgleefplan | Cliënt/Naaste | Medewerkers verzorgen mij volgens de afspraken in het zorgleefplan. |
| Verzorging volgens Zorgleefplan | Verzorgende/Verpleegkundige/Helpende | Medewerkers geven de zorg en ondersteuning die is afgesproken en opgeschreven in het zorgleefplan. |
| Medewerkers en behandelaren werken goed samen | Verzorgende/Verpleegkundige/Helpende | De zorgmedewerkers, behandelaren, specialist ouderengeneeskunde en huisarts werken goed samen. |
| Zorgleefplan 2x per jaar besproken | Cliënt/Naaste | Zorgmedewerkers bespreken het zorgleefplan minimaal twee keer per jaar met mij en/of mijn naaste. |
| Zorgleefplan 2x per jaar besproken | Verzorgende/Verpleegkundige/Helpende | Medewerkers bespreken het zorgleefplan minimaal twee keer per jaar met de bewoner en/of naasten, bijvoorbeeld in een multidisciplinair overleg. |
| Wensen en behoeften bewoners zijn leidend | Cliënt/Naaste | Medewerkers komen zoveel mogelijk tegemoet aan mijn wensen en behoeften, ook als dat betekent dat ze moeten afwijken van de afspraken in het zorgleefplan. |
| Wensen en behoeften bewoners zijn leidend | Verzorgende/Verpleegkundige/Helpende | Medewerkers komen zoveel mogelijk tegemoet aan de wensen en behoeften van de bewoners, ook als dat betekent dat ze moeten afwijken van de afspraken in het zorgleefplan. |
| Thema 2: Wonen en Welzijn | | |
| Aandacht voor levensvragen | Bestuur/(Zorg)management/Beleid en kwaliteit | Wij hebben duidelijk beleid omtrent de ondersteuning van bewoners bij levensvragen. |
| Aandacht voor levensvragen | Cliënt/Naaste | Medewerkers hebben aandacht voor mijn levensvragen. |
| Aandacht voor levensvragen | Verzorgende/Verpleegkundige/Helpende | Medewerkers hebben aandacht voor de levensvragen van bewoners. |
| Actieve bijdrage familie | Bestuur/(Zorg)management/Beleid en kwaliteit | Wij hebben duidelijk beleid omtrent familieparticipatie. |
| Actieve bijdrage familie | Cliënt/Naaste | Medewerkers vragen mijn naasten actief bij te dragen aan de zorg en ondersteuning. |
| Actieve bijdrage familie | Verzorgende/Verpleegkundige/Helpende | Medewerkers vragen familie en naasten actief bij te dragen aan de zorg en ondersteuning. |
| Persoonlijke verzorging bewoners | Cliënt/Naaste | Medewerkers hebben aandacht voor mijn persoonlijke verzorging. |
| Persoonlijke verzorging bewoners | Verzorgende/Verpleegkundige/Helpende | Medewerkers hebben aandacht voor de persoonlijke verzorging van de bewoner. |
| Hygiene: schone ruimtes | Bestuur/(Zorg)management/Beleid en kwaliteit | Wij monitoren het functioneren van onze schoonmaakdiensten. |
| Hygiene: schone ruimtes | Cliënt/Naaste | Mijn kamer en gemeenschappelijke ruimtes zijn schoon. |
| Hygiene: schone ruimtes | Verzorgende/Verpleegkundige/Helpende | De kamers van de bewoners en gemeenschappelijke ruimtes zijn schoon. |
| Zinvolle tijdsbesteding en activiteiten | Bestuur/(Zorg)management/Beleid en kwaliteit | Wij hebben duidelijk beleid omtrent zinvolle tijdsbesteding van bewoners. |
| Zinvolle tijdsbesteding en activiteiten | Cliënt/Naaste | Er zijn overdag en 's avonds voldoende activiteiten die aansluiten bij mijn behoeften en interesses. |
| Zinvolle tijdsbesteding en activiteiten | Verzorgende/Verpleegkundige/Helpende | Er zijn overdag en 's avonds voldoende activiteiten die aansluiten bij de behoeften en interesses van de bewoners. |
| Goede samenwerking vrijwilligers | Bestuur/(Zorg)management/Beleid en kwaliteit | Er is duidelijk beleid ten aanzien van het werven, inzetten en waarderen van vrijwilligers. |
| Goede samenwerking vrijwilligers | Verzorgende/Verpleegkundige/Helpende | Medewerkers werken goed samen met vrijwilligers en stemmen af wat ze van elkaar verwachten. |
| Tevredenheid maaltijden | Bestuur/(Zorg)management/Beleid en kwaliteit | Wij evalueren regelmatig de kwaliteit van de maaltijden en de tevredenheid van de bewoners omtrent de maaltijden. |
| Tevredenheid maaltijden | Cliënt/Naaste | Ik ben in het algemeen tevreden over de maaltijden. |
| Tevredenheid maaltijden | Verzorgende/Verpleegkundige/Helpende | Bewoners zijn in het algemeen tevreden over de maaltijden. |
| Leefruimtes passend bij doelgroep | Bestuur/(Zorg)management/Beleid en kwaliteit | Wij hebben ons beleid omtrent het inrichten van de leefruimtes zorgvuldig afgestemd op de behoeften van de doelgroep. |
| Leefruimtes passend bij doelgroep | Cliënt/Naaste | Mijn kamer en gemeenschappelijke ruimtes zijn zo veel mogelijk ingericht naar mijn wensen en behoeften. |
| Leefruimtes passend bij doelgroep | Verzorgende/Verpleegkundige/Helpende | De kamer en gemeenschappelijke ruimtes zijn zo veel mogelijk ingericht naar de wensen en behoeften van de bewoners. |
| Thema 3: Veilige zorg | | |
| Risico-signalering | Bestuur/(Zorg)management/Beleid en kwaliteit | Wij hebben duidelijk beleid omtrent het signaleren van risico's. |
| Risico-signalering | Cliënt/Naaste | De zorgmedewerkers zijn alert op mijn gezondheid. |
| Risico-signalering | Verzorgende/Verpleegkundige/Helpende | De zorgmedewerkers zijn alert op de gezondheid van bewoners. |
| Melden incidenten | Bestuur/(Zorg)management/Beleid en kwaliteit | Wij hebben duidelijke afspraken met medewerkers over het voorkomen en omgaan met incidenten en fouten (MIC en MIM). |
| Melden incidenten | Verzorgende/Verpleegkundige/Helpende | Zorgmedewerkers ervaren geen drempels om incidenten en fouten (MIC en MIM) te melden: het is gemakkelijk en wordt onderling en door leidinggevenden gestimuleerd. |
| Opvolgen incidenten | Bestuur/(Zorg)management/Beleid en kwaliteit | Wij hebben duidelijke afspraken met medewerkers over het melden en opvolgen van incidenten en fouten (MIC en MIM). |
| Opvolgen incidenten | Cliënt/Naaste | Incidenten en fouten worden met mij of mijn naaste besproken |
| Opvolgen incidenten | Verzorgende/Verpleegkundige/Helpende | Bij incidenten en fouten (MIC en MIM) bedenkt het team verbeteringen en voert deze uit. De bewoner en/of naasten worden van de MIC's op de hoogte gehouden. |
| Bevoegd en bekwaam | Bestuur/(Zorg)management/Beleid en kwaliteit | Wij hebben duidelijke afspraken gemaakt over welke zorgmedewerkers voorbehouden en risicovolle handelingen mogen uitvoeren. |
| Bevoegd en bekwaam | Verzorgende/Verpleegkundige/Helpende | Alleen bevoegde en bekwame zorgmedewerkers voeren voorbehouden en risicovolle handelingen uit. |
| Bevoegd en bekwaam | Bestuur/(Zorg)management/Beleid en kwaliteit | Wij hebben duidelijk beleid omtrent het bewaren en verstrekken van medicijnen. |
| Medicatieveiligheid | Verzorgende/Verpleegkundige/Helpende | Medicijnen worden bewaard en verstrekt volgens de gemaakte afspraken. |
| Zorg en dwang | Bestuur/(Zorg)management/Beleid en kwaliteit | Wij hebben duidelijk beleid omtrent vrijheidsbeperkende maatregelen en onvrijwillige zorg. |
| Zorg en dwang | Verzorgende/Verpleegkundige/Helpende | Medewerkers weten wat ze moeten doen om vrijheidsbeperkende maatregelen te voorkomen en op welke wijze zij deze kunnen inzetten (als ze toch nodig zijn). |
| Medewerkers werken hygienisch | Bestuur/(Zorg)management/Beleid en kwaliteit | Wij hebben duidelijk beleid omtrent hygiënisch werken. |
| Medewerkers werken hygienisch | Cliënt/Naaste | Medewerkers werken hygiënisch en netjes. |
| Medewerkers werken hygienisch | Verzorgende/Verpleegkundige/Helpende | Medewerkers werken hygiënisch en netjes. |
| Voedselveiligheid | Bestuur/(Zorg)management/Beleid en kwaliteit | Wij hebben duidelijk beleid omtrent voedselveiligheid (HACCP). |
| Voedselveiligheid | Verzorgende/Verpleegkundige/Helpende | Medewerkers werken volgens de richtlijnen voor voedselveiligheid (HACCP). |
| Thema 4: Leren en ontwikkelen | | |
| Reflectie en bespreken wat beter kan | Bestuur/(Zorg)management/Beleid en kwaliteit | Wij stimuleren een open cultuur waarin medewerkers reflecteren op het werk en bespreken met elkaar wat beter kan. |
| Reflectie en bespreken wat beter kan | Cliënt/Naaste | Ik merk dat medewerkers nadenken over hoe hun werk beter kan. |
| Reflectie en bespreken wat beter kan | Verzorgende/Verpleegkundige/Helpende | Medewerkers denken kritisch na over hoe hun werk beter kan en bespreken dat met elkaar. >> naar thema Veiligheid? |
| Voldoende overlegmomenten | Bestuur/(Zorg)management/Beleid en kwaliteit | Wij bieden medewerkers voldoende tijd voor werkoverleg. |
| Voldoende overlegmomenten | Verzorgende/Verpleegkundige/Helpende | Wij hebben voldoende overlegmomenten om belangrijke zaken te bespreken en af te stemmen. |
| Zorgmedewerkers kunnen input geven voor kwaliteitsplan | Bestuur/(Zorg)management/Beleid en kwaliteit | De organisatie beschikt over een actueel kwaliteitsplan en -verslag dat is opgesteld in afstemming met cliënten, naasten, cliëntenraad en medewerkers. |
| Zorgmedewerkers kunnen input geven voor kwaliteitsplan | Verzorgende/Verpleegkundige/Helpende | Medewerkers kunnen ideeën inbrengen voor het kwaliteitsplan en -verslag van onze organisatie. |
| Kwaliteitsmanagementsysteem, procedures | Bestuur/(Zorg)management/Beleid en kwaliteit | Wij hebben een kwaliteitsmanagementsysteem dat ondersteunend is aan kwaliteitsborging en verbetering. |
| Kwaliteitsmanagementsysteem, procedures | Verzorgende/Verpleegkundige/Helpende | De organisatie heeft werkwijzen, procedures en richtlijnen opgesteld die medewerkers gebruiken bij hun dagelijkse werkzaamheden. |
| Cultuur van leren en verbeteren | Bestuur/(Zorg)management/Beleid en kwaliteit | Er zijn duidelijke afspraken ten aanzien van het leren en verbeteren van de kwaliteit van zorg en wij ondersteunen medewerkers zodat zij hiernaar kunnen handelen. |
| Cultuur van leren en verbeteren | Cliënt/Naaste | Medewerkers nemen mijn opmerkingen en tips serieus en koppelen terug wat ermee is gedaan. |
| Cultuur van leren en verbeteren | Verzorgende/Verpleegkundige/Helpende | Wij leren van situaties en (cliënt)ervaringen en koppelen terug aan bewoners, naasten en elkaar wat ermee is gedaan. |
| Voldoende tijd en ruimte voor deelname aan lerend netwer | Bestuur/(Zorg)management/Beleid en kwaliteit | Wij hebben een goed functionerend lerend netwerk en scheppen voorwaarden zodat er voor alle medewerkers tijd en ruimte beschikbaar is om mee te lopen en kennis te delen bij een collega-organisatie. |
| Voldoende tijd en ruimte voor deelname aan lerend netwer | Verzorgende/Verpleegkundige/Helpende | Medewerkers krijgen voldoende tijd om zelf mee te lopen met andere collega's of organisaties (in een lerend netwerk) om van elkaar te leren. |
| Thema 5: Leiderschap, management en governance | | |
| Organisatie heeft heldere visie | Bestuur/(Zorg)management/Beleid en kwaliteit | De organisatie heeft een heldere visie op waar de organisatie voor gaat en staat. |
| Organisatie heeft heldere visie | Cliënt/Naaste | Ik merk dat medewerkers volgens de visie van de organisatie werken, zij weten waar de organisatie voor gaat en staat. |
| Organisatie heeft heldere visie | Verzorgende/Verpleegkundige/Helpende | Medewerkers werken volgens de visie van de organisatie, zij weten waar de organisatie voor gaat en staat. |
| Bestuur en MT sturen op kwaliteit | Bestuur/(Zorg)management/Beleid en kwaliteit | De kwaliteit van zorg en persoonsgerichte zorg zijn belangrijke thema's voor het bestuur en het management. |
| Bestuur en MT sturen op kwaliteit | Verzorgende/Verpleegkundige/Helpende | Ik merk dat de kwaliteit van zorg en persoonsgerichte zorg belangrijke thema's zijn voor het bestuur en management. |
| Leiding is betrokken | Bestuur/(Zorg)management/Beleid en kwaliteit | Het bestuur en management is betrokken en weet wat er speelt op de locatie. |
| Leiding is betrokken | Verzorgende/Verpleegkundige/Helpende | Het bestuur en management is betrokken en weet wat er speelt op de locatie. |
| Leiding is ondersteunend | Bestuur/(Zorg)management/Beleid en kwaliteit | Het bestuur en management weten wat medewerkers nodig hebben om hun werk goed uit te kunnen voeren en ondersteunen hen hierin. |
| Leiding is ondersteunend | Verzorgende/Verpleegkundige/Helpende | Het bestuur en management weten wat medewerkers nodig hebben om hun werk goed uit te kunnen voeren en ondersteunen hen hierin. |
| Invloed uitoefenen op beleid | Bestuur/(Zorg)management/Beleid en kwaliteit | Wij maken gebruik van de adviezen die voortkomen uit de verschillende adviesraden zoals de VAR, PAR, (C)CR en OR. |
| Invloed uitoefenen op beleid | Cliënt/Naaste | Ik kan invloed uitoefenen op het beleid van de organisatie via de cliëntenraad (CR). |
| Invloed uitoefenen op beleid | Verzorgende/Verpleegkundige/Helpende | Ik kan invloed uitoefenen op het beleid van de organisatie via de verpleegkundige/verzorgende adviesraad (VAR). |
| Thema 6: Personeelssamenstelling | | |
| Deskundig personeel: voldoende kennis en vaardigheden | Bestuur/(Zorg)management/Beleid en kwaliteit | Wij maken minimaal één keer per jaar een strategische personeelsplanning om het gewenste zorgaanbod te realiseren. |
| Deskundig personeel: voldoende kennis en vaardigheden | Cliënt/Naaste | De zorgmedewerkers zijn deskundig en hebben voldoende kennis en vaardigheden voor het bieden van goede zorg. |
| Deskundig personeel: voldoende kennis en vaardigheden | Verzorgende/Verpleegkundige/Helpende | De zorgmedewerkers zijn deskundig en hebben voldoende kennis en vaardigheden om goede zorg te bieden die past bij de veranderende zorgvraag van bewoners. |
| Voldoende personeel voor uitvoering van de zorg | Bestuur/(Zorg)management/Beleid en kwaliteit | Wij zetten voldoende zorgmedewerkers in om de dagelijkse zorg goed uit te voeren en om over deze zorg te rapporteren. |
| Voldoende personeel voor uitvoering van de zorg | Cliënt/Naaste | Er worden voldoende zorgmedewerkers ingezet om mij goed te kunnen verzorgen en ondersteunen. |
| Voldoende personeel voor uitvoering van de zorg | Verzorgende/Verpleegkundige/Helpende | Er worden voldoende zorgmedewerkers ingezet om de dagelijkse zorg goed uit te voeren en om over deze zorg te rapporteren. |
| Intensieve zorgmomenten minimaal twee zorgmedewerkers | Bestuur/(Zorg)management/Beleid en kwaliteit | Wij zorgen ervoor dat er bij intensieve zorgmomenten minimaal twee zorgmedewerkers beschikbaar zijn om de taken te verrichten. |
| Intensieve zorgmomenten minimaal twee zorgmedewerkers | Verzorgende/Verpleegkundige/Helpende | Bij intensieve zorgmomenten die meer aandacht vragen zijn er minimaal twee zorgmedewerkers beschikbaar om de taken te verrichten. |
| Altijd iemand in de huiskamer of gemeenschappelijke ruimte | Bestuur/(Zorg)management/Beleid en kwaliteit | Wij zorgen ervoor dat er overdag en 's avonds altijd iemand in de gemeenschappelijke ruimte aanwezig is om bewoners aandacht en nabijheid te bieden en toezicht te houden. |
| Altijd iemand in de huiskamer of gemeenschappelijke ruimte | Cliënt/Naaste | Mijn ervaring is dat er overdag en 's avonds altijd iemand in de huiskamer of gemeenschappelijke ruimte aanwezig is om mij aandacht te geven. |
| Altijd iemand in de huiskamer of gemeenschappelijke ruimte | Verzorgende/Verpleegkundige/Helpende | Overdag en 's avonds is er altijd iemand in de huiskamer of gemeenschappelijke ruimte aanwezig om bewoners aandacht en nabijheid te bieden en toezicht te houden. |
| Vast personeel | Bestuur/(Zorg)management/Beleid en kwaliteit | De bewoners worden meestal geholpen door medewerkers die de bewoners kennen (vast personeel). |
| Vast personeel | Cliënt/Naaste | Ik word meestal geholpen door medewerkers die ik ken (vast personeel). |
| Vast personeel | Verzorgende/Verpleegkundige/Helpende | De bewoners worden meestal geholpen door medewerkers die de bewoners kennen (vast personeel). |
| Goede samenwerking in het team | Cliënt/Naaste | Ik merk dat medewerkers goed met elkaar samenwerken. |
| Goede samenwerking in het team | Verzorgende/Verpleegkundige/Helpende | Medewerkers werken goed met elkaar samen. Ze informeren elkaar over wat er is gebeurd of is afgesproken. |
| Scholing, sluit aan bij behoefte | Bestuur/(Zorg)management/Beleid en kwaliteit | Wij zorgen ervoor dat scholing aansluit op de ontwikkel- en leerbehoeften van onze medewerkers. |
| Scholing, sluit aan bij behoefte | Verzorgende/Verpleegkundige/Helpende | Er wordt voldoende scholing georganiseerd die aansluit bij de behoefte van medewerkers en bij de veranderende zorgvraag van bewoners. |
| Medewerkers voldoende tijd voor scholing | Bestuur/(Zorg)management/Beleid en kwaliteit | Wij zorgen ervoor dat medewerkers voldoende tijd krijgen voor persoonlijke ontwikkeling en scholing. |
| Medewerkers voldoende tijd voor scholing | Verzorgende/Verpleegkundige/Helpende | Medewerkers krijgen voldoende tijd om scholing te volgen en nieuwe competenties en vaardigheden te leren. |
| Jaarlijks functioneringsgesprek | Bestuur/(Zorg)management/Beleid en kwaliteit | Medewerkers hebben jaarlijks een functioneringsgesprek waarin ook persoonlijke ontwikkeldoelen en scholing worden besproken. |
| Jaarlijks functioneringsgesprek | Verzorgende/Verpleegkundige/Helpende | Medewerkers hebben periodiek een functioneringsgesprek waarin ook persoonlijke ontwikkeldoelen en scholing worden besproken. |
| Ziekteverzuim | Bestuur/(Zorg)management/Beleid en kwaliteit | Medewerkers zijn zelden afwezig door (ziekte)verzuim en dit vormt geen structureel probleem. |
| Ziekteverzuim | Verzorgende/Verpleegkundige/Helpende | Medewerkers zijn zelden afwezig door (ziekte)verzuim en dit vormt geen structureel probleem. |
| Verloop van personeel | Bestuur/(Zorg)management/Beleid en kwaliteit | Er zijn weinig wisselingen in de teams en het verloop van medewerkers vormt geen structureel probleem. |
| Verloop van personeel | Verzorgende/Verpleegkundige/Helpende | Er zijn weinig wisselingen in de teams en het verloop van medewerkers vormt geen structureel probleem. |
| Met plezier naar werk | Bestuur/(Zorg)management/Beleid en kwaliteit | Ik ga met plezier naar mijn werk. |
| Met plezier naar werk | Verzorgende/Verpleegkundige/Helpende | Ik ga met plezier naar mijn werk. |
| Thema 7: Gebruik van hulpbronnen | | |
| Regelmatige evaluatie werkwijzen | Bestuur/(Zorg)management/Beleid en kwaliteit | De werkwijzen en procedures worden regelmatig geëvalueerd en aangepast als dat nodig is. |
| Regelmatige evaluatie werkwijzen | Verzorgende/Verpleegkundige/Helpende | Medewerkers evalueren regelmatig de werkwijzen en procedures en passen deze aan waar nodig. |
| Vindbaarheid materialen en hulpmiddelen | Verzorgende/Verpleegkundige/Helpende | De materialen en hulpmiddelen zijn altijd te vinden op de afgesproken plek. |
| Mogelijkheden voor inzet (vernieuwende) technologie | Bestuur/(Zorg)management/Beleid en kwaliteit | Wij ondersteunen en stimuleren medewerkers bij het gebruik van technologie. |
| Mogelijkheden voor inzet (vernieuwende) technologie | Verzorgende/Verpleegkundige/Helpende | Zorgmedewerkers worden gestimuleerd om technologie (e-health) te gebruiken bij de zorgverlening. |
| Facilitaire diensten ondersteunen zorgmedewerkers | Bestuur/(Zorg)management/Beleid en kwaliteit | De organisatie is zo ingericht dat de facilitaire diensten ondersteunend zijn aan de zorgteams om goede zorg te kunnen leveren. |
| Facilitaire diensten ondersteunen zorgmedewerkers | Verzorgende/Verpleegkundige/Helpende | De facilitaire diensten (keuken, technische dienst en huishouding) zijn ondersteunend aan de zorgteams. |
| Ondersteunende diensten ondersteunen zorgproces | Bestuur/(Zorg)management/Beleid en kwaliteit | De organisatie is zo ingericht dat de ondersteunende diensten ondersteunend zijn aan de zorgteams om goede zorg te kunnen verlenen. |
| Ondersteunende diensten ondersteunen zorgproces | Verzorgende/Verpleegkundige/Helpende | De ondersteunende diensten (P&O, kwaliteit, administratie en financiën) zijn ondersteunend aan de zorgteams. |
| Thema 8: Gebruik van informatie | | |
| Cliëntervaringen minimaal 1 keer per jaar gemeten | Bestuur/(Zorg)management/Beleid en kwaliteit | Wij vragen bewoners minimaal één keer per jaar naar hun ervaringen met de zorg, bijvoorbeeld via een cliëntervaringsonderzoek of interview. |
| Cliëntervaringen minimaal 1 keer per jaar gemeten | Cliënt/Naaste | Minimaal één keer per jaar word ik gevraagd naar mijn ervaringen met de zorg, bijvoorbeeld via een cliëntervaringsonderzoek of interview. |
| Cliëntervaringen minimaal 1 keer per jaar gemeten | Verzorgende/Verpleegkundige/Helpende | Minimaal één keer per jaar worden bewoners of naasten gevraagd naar de ervaringen met de zorg, bijvoorbeeld via een cliëntervaringsonderzoek of interview. |
| Gebruik cliëntervaringen om zorg te verbeteren | Bestuur/(Zorg)management/Beleid en kwaliteit | In onze organisatie vormen cliëntervaringsonderzoeken en andere vormen van cliëntfeedback belangrijke informatie voor het verbeteren van de zorgverlening. |
| Gebruik cliëntervaringen om zorg te verbeteren | Cliënt/Naaste | Medewerkers maken gebruik van de tips en adviezen uit het cliëntervaringsonderzoek om de zorgverlening te verbeteren. |
| Gebruik cliëntervaringen om zorg te verbeteren | Verzorgende/Verpleegkundige/Helpende | Medewerkers maken gebruik van de tips en adviezen uit het cliëntervaringsonderzoek om de zorgverlening te verbeteren. |
| (Stuur)informatie is beschikbaar o.a. t.b.v. leren en verbeteren | Bestuur/(Zorg)management/Beleid en kwaliteit | Wij beschikken over actuele en betrouwbare informatie over de kwaliteit van zorg en sturen bij op mogelijke risico's. |
| (Stuur)informatie is beschikbaar o.a. t.b.v. leren en verbeteren | Verzorgende/Verpleegkundige/Helpende | Medewerkers hebben toegang tot informatie over incidenten, cliëntervaringen en audits om de zorg te kunnen verbeteren. |
| Administratieve lasten | Bestuur/(Zorg)management/Beleid en kwaliteit | Wij zijn alert op onnodige administratie en proberen de regeldruk in de organisatie te verlagen. |
| Administratieve lasten | Verzorgende/Verpleegkundige/Helpende | De organisatie probeert de administratieve lasten voor medewerkers zoveel mogelijk te beperken. |
